# Association of systemic inflammation response index and triglyceride-glucose index with the severity of coronary artery stenosis in elderly patients: a retrospective cross-sectional study

**DOI:** 10.3389/fcvm.2026.1809166

**Published:** 2026-05-18

**Authors:** Yazhao Sun, Xiao Yu

**Affiliations:** 1 Department of Cardiology, Cangzhou People's Hospital, Cangzhou, Hebei, China; 2 Department of Neurology Intervention, Cangzhou People's Hospital, Cangzhou, Hebei, China

**Keywords:** coronary artery disease, coronary artery stenosis, insulin resistance, systemic inflammation response index, triglyceride-glucose index

## Abstract

**Background:**

Atherosclerosis is significantly influenced by chronic inflammation and insulin resistance (IR). This study aims to explore the effects of the Systemic Inflammation Response Index (SIRI) and the Triglyceride-Glucose Index (TyG) on the severity of coronary artery stenosis in patients with coronary artery disease (CAD).

**Methods:**

This study is a retrospective cross-sectional study conducted at a single center, including 1,019 CAD patients. Based on the median Gensini score, patients were divided into low stenosis (Gensini score < 30) and high stenosis (Gensini score ≥ 30) groups. Logistic regression, interaction analysis, receiver operating characteristic (ROC) curve, Spearman correlation analysis, mediation analysis, and sensitivity analysis using Gensini score tertiles were used to explore the relationship between SIRI, TyG, and the severity of coronary artery stenosis.

**Results:**

Multivariable logistic regression analysis revealed that SIRI (OR: 1.231, 95% CI: 1.025–1.489, *P* = 0.006) and TyG (OR: 2.458, 95% CI: 1.737–3.502, *P* < 0.001) were significantly associated with severe stenosis. Based on the median values of SIRI (0.961) and TyG (8.620), participants were divided into four groups, with the low SIRI + low TyG group as the reference. The OR for the high SIRI + high TyG group was 4.912 (95% CI: 3.185–7.649, *P* < 0.001). A significant additive interaction between SIRI and TyG was observed, with RERI: 1.947 (95% CI: 0.208–4.103), API: 0.396 (95% CI: 0.042–0.616), and S: 1.991 (95% CI: 1.067–4.430). The inclusion of SIRI and TyG significantly improved predictive performance compared to the traditional risk model, with a Net Reclassification Improvement (NRI) of 0.334 (*P* < 0.001) and an Integrated Discrimination Improvement (IDI) of 0.036 (*P* < 0.001). Spearman correlation analysis confirmed significant positive correlations of SIRI (*ρ* = 0.435, *P* < 0.001) and TyG (*ρ* = 0.347, *P* < 0.001) with the continuous Gensini score. In the mediation analysis, TyG accounted for 46.7% of the association between SIRI and severe stenosis via a significant indirect statistical association, while SIRI accounted for 21.3% of the association between TyG and severe stenosis via a significant indirect statistical association. Sensitivity analysis using Gensini tertiles confirmed that both SIRI (OR: 1.371, 95% CI: 1.181–1.591, *P* < 0.001) and TyG (OR: 1.491, 95% CI: 1.181–1.883, *P* < 0.001) remained independently associated with higher stenosis severity.

**Conclusion:**

SIRI and TyG are important indicators for predicting the risk of coronary artery stenosis, with an interaction between them, and they exhibit significant indirect statistical associations with each other.

## Introduction

Cardiovascular diseases are the leading causes of death and disability worldwide, with coronary artery disease (CAD) being the most common clinical type. According to the 2019 Global Burden of Disease Study, the global prevalence of cardiovascular diseases increased from approximately 271 million cases in 1990 to around 523 million cases in 2019, accompanied by a significant rise in mortality, reflecting the ongoing impact of population growth, aging, and increased exposure to risk factors on the global cardiovascular burden (1). Atherosclerosis is the primary pathological basis of CAD, characterized by chronic, multifactorial inflammation centered on lipid deposition. In this process, low-density lipoprotein, rich in apolipoprotein B, accumulates and oxidizes in the vascular endothelium, triggering endothelial dysfunction, immune cell recruitment, chronic inflammation, oxidative stress, and apoptosis, ultimately promoting plaque formation, progression, and instability (2–4).

Inflammation has emerged as a key driver of plaque formation and instability in atherosclerosis (5). Previous studies have shown that neutrophils, monocytes, and lymphocytes, components of white blood cells, serve as reliable biomarkers of systemic inflammation (6). During the inflammatory process of atherosclerosis, neutrophils and monocytes are recruited to the site of inflammation, activating lipid-laden macrophages, while lymphocytes play immune-regulatory and protective roles (7). In 2016, Qi et al. introduced the Systemic Inflammation Response Index (SIRI), which integrates neutrophil, monocyte, and lymphocyte counts from peripheral blood, providing a comprehensive reflection of inflammation pathways and immune dysregulation (8). In the general population, elevated SIRI levels have been associated with an increased risk of carotid atherosclerosis, suggesting its potential clinical value in assessing atherosclerosis (9). Liuizė et al. confirmed the importance of SIRI in CAD risk assessment, with higher SIRI levels observed in patients with higher Gensini scores (10). Additionally, several studies have demonstrated that SIRI can be used to evaluate prognosis in various diseases, including malignancies, rheumatoid arthritis, heart failure, myocardial infarction, and hypertension (11–15).

Recent studies have shown that metabolic abnormalities, particularly insulin resistance (IR), not only accelerate inflammation by worsening dyslipidemia and endothelial damage but also independently predict plaque progression, acting as a critical bridge in the inflammation-metabolism interaction, which further promotes the development of atherosclerosis (16). IR has been recognized as a new risk factor for CAD. However, the gold standard for diagnosing IR is the high insulin-normal glucose clamp test, which is time-consuming and labor-intensive, limiting its application in large-scale studies. In 2008, Guerrero-Romero first introduced the triglyceride-glucose index (TyG) as an alternative marker for IR (17). Furthermore, the TyG has been shown to be an important marker for atherosclerosis. IR-induced vascular damage includes functional and structural changes in the arterial wall, such as limited vasodilation, arterial stiffness, vascular calcification, and thickening of the arterial wall (18). Irace et al. found that after adjusting for traditional cardiovascular risk factors, the TyG was significantly associated with carotid atherosclerosis (19). A recent study from Korea reported that the TyG was associated with an increased risk of coronary artery stenosis in patients with type 2 diabetes based on coronary computed tomography angiography (20). A series of studies has also confirmed the clinical value of the TyG in predicting adverse cardiovascular events (21–23). However, there is a lack of in-depth research on the interaction between inflammation and IR in coronary artery stenosis. Given that inflammation and IR synergistically contribute to the progression of metabolic diseases and adverse events (24), we aim to explore the effects of SIRI and TyG on the degree of coronary artery stenosis in CAD patients. This study uses invasive coronary angiography to calculate the Gensini score for assessing the severity of coronary atherosclerosis. The findings not only help in the early identification of high-risk patients but also provide guidance for subsequent intervention strategies, ultimately improving the treatment outcomes and quality of life of CAD patients.

## Methods

### Study population

This retrospective cross-sectional study included 1,019 patients diagnosed with CAD at Cangzhou People's Hospital between July and December 2024. The inclusion criteria were: (1) age ≥ 60 years; and (2) patients hospitalized with angina pectoris (stable or unstable) who underwent elective coronary angiography for evaluation of suspected coronary artery disease. The exclusion criteria were: (1) acute myocardial infarction (including STEMI and NSTEMI) as the index presentation during the current hospitalization; (2) a history of CAD, prior percutaneous coronary intervention, or coronary artery bypass grafting; (3) current or recent (within 3 months prior to admission) use of lipid-lowering medications (including statins, ezetimibe, PCSK9 inhibitors, or fibrates); (4) missing key clinical data such as fasting blood glucose (FBG), triglycerides (TG), neutrophils, lymphocytes, or monocytes; (5) absence of coronary angiography (CAG) records; (6) severe inflammatory or autoimmune diseases (e.g., rheumatoid arthritis, systemic lupus erythematosus, or active infections); (7) malignancies or severe liver or kidney dysfunction; (8) hereditary hyperlipidemia; (9) severe hematologic diseases (e.g., aplastic anemia, leukemia, or lymphoma); and (10) missing other essential clinical data. This study was approved by the Ethics Committee of Cangzhou People's Hospital (Approval No. K2025-130-02), which specifically granted a waiver of informed consent due to the retrospective cross-sectional nature of the study, the exclusive use of de-identified data collected during routine clinical care, and the absence of any risk to participants.

### Grouping

Based on the median Gensini score, patients were divided into two groups: the mild stenosis group (Gensini score < 30, *n* = 496) and the severe stenosis group (Gensini score ≥ 30, *n* = 523). The median value was used as the cutoff for grouping based on the following considerations. Currently, there is no universally accepted clinical threshold for defining severe stenosis using the Gensini score; therefore, a data-driven approach was adopted for grouping. This method ensures balanced sample sizes between the two groups, enhances statistical power, and minimizes bias associated with arbitrary threshold selection. It is a commonly used approach in exploratory studies investigating risk factors associated with the severity of coronary artery stenosis. The Gensini score is a widely utilized scoring system in CAG for assessing the severity of CAD (25). This scoring system assigns points to each coronary lesion according to the degree of stenosis, as follows: ≤25% stenosis is assigned 1 point, 26%–50% is assigned 2 points, 51%–75% is assigned 4 points, 76%–90% is assigned 8 points, 91%–99% is assigned 16 points, and total occlusion (100%) is assigned 32 points, reflecting the severity of the stenosis. In addition, the Gensini score incorporates the anatomical location of the lesions, with different weight coefficients assigned to various coronary branches. The left main coronary artery (LMCA) is assigned the highest weight of 5 points; the proximal left anterior descending artery (LAD) is assigned 2.5 points, the mid LAD is assigned 1.5 points, and the distal LAD is assigned 1 point. The right coronary artery (RCA), left circumflex artery (LCX), and smaller branches are assigned lower weight coefficients (1 point or 0.5 points), reflecting the significance of each coronary branch in supplying blood to the heart. By integrating the degree of stenosis and the weight of lesion location, the Gensini score provides a comprehensive evaluation of the extent of coronary artery lesions, offering valuable insights for clinical risk assessment.

### Data collection

This study collected clinical data from patients, including demographic characteristics, clinical history, and laboratory parameters. Demographic characteristics included age, sex, body mass index (BMI), and smoking history. Clinical history included atrial fibrillation, chronic obstructive pulmonary disease (COPD), hypertension, and diabetes, and medication history. Medication history, encompassing the use of lipid-lowering, antidiabetic, and antiplatelet agents, was obtained through review of electronic medical records and patient self-report at the time of admission. Laboratory parameters covered various common biochemical markers, including FBG, glycated hemoglobin (HbA1c), albumin, alanine aminotransferase (ALT), aspartate aminotransferase (AST), creatinine, uric acid, D-Dimer, TG, total cholesterol (TC), low-density lipoprotein cholesterol (LDL-C), high-density lipoprotein cholesterol (HDL-C), C-reactive protein (CRP), leukocytes, erythrocytes, platelets, hemoglobin, monocytes, lymphocytes, and neutrophils. Additionally, the TyG was calculated as Ln [TG (mg/dL) × FBG (mg/dL)/2], and the SIRI was calculated as neutrophils × monocytes/lymphocytes.

### Statistical analysis

All statistical analyses in this study were performed using R software (version 4.4.2). Continuous variables were expressed as mean ± standard deviation (SD) for normally distributed data or median (interquartile range, IQR) for non-normally distributed data. Group comparisons were conducted using the T-test or Mann–Whitney U test, depending on the distribution of the data. Categorical variables were presented as counts and percentages, and group comparisons were made using the chi-square test or Fisher's exact test. Spearman correlation analysis was performed to assess the associations between SIRI, TyG, and the continuous Gensini score. Logistic regression analysis was used to evaluate the independent association between SIRI and TyG indices and the risk of severe stenosis, adjusting for confounding factors including age, BMI, smoking, hypertension, diabetes, HbA1c, LDL-C, HDL-C, creatinine, leukocytes, uric acid, CRP, and fibrinogen. Prior to constructing Model 2, collinearity diagnostics were performed on all candidate covariates, and the results showed that the variance inflation factor for all variables was less than 5, indicating no severe multicollinearity. Participants were divided into four groups based on the median values of SIRI (0.961) and TyG (8.620) to assess their association with severe stenosis. The additive interaction of SIRI and TyG was assessed by calculating the Relative Excess Risk of Interaction (RERI), Attributable Proportion of Interaction (API), and Synergy Index (S). Receiver operating characteristic (ROC) curves were used to analyze model performance, and the area under the curve (AUC) was calculated. Additionally, the Net Reclassification Improvement (NRI) and Integrated Discrimination Improvement (IDI) were computed to evaluate the incremental predictive value of SIRI and TyG beyond traditional risk factors. Mediation analysis was performed to explore the reciprocal mediation effects of SIRI and TyG in their relationship with severe stenosis. A sensitivity analysis was performed using ordinal logistic regression with Gensini score tertiles as the outcome to assess the robustness of the findings to alternative categorization of stenosis severity. All statistical tests were two-sided, and *P* values < 0.05 were considered statistically significant.

## Results

### Participant characteristics

According to the inclusion and exclusion criteria, a total of 1,019 elderly participants were included in the final analysis. Based on the degree of coronary artery stenosis, participants were classified into the Mild stenosis group (*n* = 496) and the Severe stenosis group (*n* = 523). The baseline characteristics of the participants are presented in Table 1. The results showed that the TyG and SIRI levels were significantly higher in the Severe stenosis group compared to the Mild stenosis group. Additionally, the Severe stenosis group had significantly higher rates of smoking, hypertension, and diabetes. The Severe stenosis group also had higher levels of age, BMI, Gensini score, FBG, HbA1c, creatinine, uric acid, fibrinogen, TG, TC, LDL-C, CRP, leukocytes, monocytes, and neutrophils than the Mild stenosis group. However, HDL-C and lymphocyte levels were lower in the Severe stenosis group. No significant differences were observed in other parameters.

**Table 1:** Baseline characteristics of the patients studied.

Characteristics	Total population	Mild stenosis	Severe stenosis	*P* value
	(*n* = 1,019)	(*n* = 496)	(*n* = 523)	
Age, years, median (IQR)	65 (63, 68)	65 (63, 68)	65 (63, 69)	0.007
Male, *n* (%)	628 (61.6)	309 (62.3)	319 (61)	0.716
BMI, kg/m^2^, median (IQR)	24.86 (23.73, 26.15)	24.72 (23.62, 25.89)	24.9 (23.8, 26.42)	0.004
Smoking, *n* (%)	250 (24.5)	86 (17.3)	164 (31.4)	<0.001
Atrial Fibrillation, *n* (%)	30 (2.9)	9 (1.8)	21 (4)	0.059
COPD, *n* (%)	58 (5.7)	22 (4.4)	36 (6.9)	0.121
Hypertension, *n* (%)	604 (59.3)	232 (46.8)	372 (71.1)	<0.001
Diabetes, *n* (%)	277 (27.2)	92 (18.5)	185 (35.4)	<0.001
Gensini Score, median (IQR)	30 (24, 38)	24 (18, 25)	38 (33, 42)	<0.001
FBG, mmol/L, median (IQR)	5.47 (4.99, 6.13)	5.39 (4.91, 5.88)	5.56 (5.07, 6.34)	<0.001
HbA1c, %, median (IQR)	5.8 (5.6, 6.1)	5.8 (5.5, 6)	5.9 (5.6, 6.2)	<0.001
Albumin, g/L, median (IQR)	43.1 (40.9, 45.3)	43.2 (41, 45.1)	43 (40.9, 45.45)	0.761
ALT, U/L, median (IQR)	19 (14, 27)	19 (14, 27)	20 (14, 27)	0.689
AST, U/L, median (IQR)	21 (17, 26)	21 (18, 26)	21 (17, 26)	0.255
Creatinine, μmol/L, median (IQR)	61.7 (52, 71.7)	58.7 (50, 70)	64 (55.1, 74)	<0.001
Uric Acid, μmol/L, median (IQR)	299 (246, 360)	289 (238.75, 354.25)	309 (254.5, 366)	0.002
D-Dimer, μg/mL, median (IQR)	0.24 (0.18, 0.33)	0.24 (0.18, 0.32)	0.25 (0.18, 0.33)	0.689
Fibrinogen, g/L, median (IQR)	3.35 (2.88, 3.83)	3.29 (2.84, 3.76)	3.43 (2.93, 3.9)	0.005
TG, mmol/L, median (IQR)	1.25 (0.91, 1.72)	1.12 (0.8, 1.58)	1.37 (1.06, 1.88)	<0.001
TC, mmol/L, median (IQR)	4.34 (3.7, 5.11)	4.11 (3.51, 4.81)	4.58 (3.91, 5.38)	<0.001
LDL-C, mmol/L, median (IQR)	2.64 (2.06, 3.27)	2.41 (1.93, 2.98)	2.86 (2.22, 3.45)	<0.001
HDL-C, mmol/L, median (IQR)	1.24 (1.03, 1.45)	1.26 (1.05, 1.47)	1.21 (1.02, 1.42)	0.020
CRP, mg/L, median (IQR)	1.94 (1.47, 2.65)	1.9 (1.33, 2.59)	2 (1.61, 2.77)	<0.001
SIRI, median (IQR)	0.96 (0.55, 1.72)	0.61 (0.46, 1.21)	1.21 (0.81, 2.36)	<0.001
TyG, median (IQR)	8.62 (8.29, 8.96)	8.46 (8.1, 8.79)	8.78 (8.45, 9.13)	<0.001
Leukocytes, 10^9^/L, median (IQR)	6.06 (5.09, 7.4)	5.72 (4.86, 6.9)	6.39 (5.28, 7.71)	<0.001
Erythrocytes, 10^12^/L, mean ± SD	4.59 ± 0.45	4.58 ± 0.45	4.59 ± 0.46	0.628
Platelets, 10^9^/L, median (IQR)	220 (184.5, 256)	220 (185, 254)	219 (184, 258.5)	0.811
Hemoglobin, g/L, median (IQR)	140 (130, 149)	139.5 (129, 148)	140 (131, 150)	0.332
Monocytes, 10^9^/L, median (IQR)	0.36 (0.28, 0.47)	0.33 (0.26, 0.42)	0.39 (0.31, 0.5)	<0.001
Lymphocytes, 10^9^/L, median (IQR)	1.46 (1.11, 1.87)	1.6 (1.26, 2.04)	1.34 (1.02, 1.69)	<0.001
Neutrophils, 10^9^/L, median (IQR)	3.94 (3.1, 5.13)	3.5 (2.79, 4.46)	4.37 (3.52, 5.6)	<0.001

BMI, body mass index; COPD, chronic obstructive pulmonary disease; FBG, fasting blood glucose; HbA1c, glycated hemoglobin; ALT, alanine aminotransferase; AST, aspartate aminotransferase; TG, triglyceride; TC, total cholesterol; LDL-C, low-density lipoprotein cholesterol; HDL-C, high-density lipoprotein cholesterol; CRP, c-reactive protein; TyG, triglyceride-glucose index; SIRI, systemic inflammation response index.

### Correlation between SIRI, TyG, and continuous Gensini score

Spearman correlation analysis further confirmed that both SIRI (*ρ* = 0.435, 95% CI: 0.384–0.483, *P* < 0.001) and TyG (*ρ* = 0.347, 95% CI: 0.292–0.400, *P* < 0.001) were significantly positively correlated with the continuous Gensini score (Supplementary Table S1), indicating that higher levels of these indices were associated with greater coronary stenosis severity on a continuous scale.

### Association between SIRI, TyG, and severe stenosis

We performed binary logistic regression analyses to assess the relationship between SIRI, TyG, and the degree of coronary artery stenosis, using two models (Table 2). Model 1 was unadjusted, while Model 2 adjusted for potential confounders such as age, BMI, smoking, hypertension, diabetes, HbA1c, LDL-C, HDL-C, creatinine, leukocytes, uric acid, CRP, and fibrinogen. The analysis revealed significant associations between both SIRI and TyG with severe stenosis. In Model 1, the OR for SIRI was 1.293 (95% CI: 1.111–1.514, *P* = 0.001), while for TyG, the OR was 3.179 (95% CI: 2.335–4.364, *P* < 0.001). In Model 2, after adjusting for confounders, the OR for SIRI was 1.231 (95% CI: 1.025–1.489, *P* = 0.006), and for TyG, it was 2.458 (95% CI: 1.737–3.502, *P* < 0.001).

**Table 2:** OR (95% CI) of SIRI and TyG for severe stenosis.

Variable	Model 1		Model 2	
	OR (95% CI)	*P* value	OR (95% CI)	*P* value
Severe stenosis				
SIRI	1.293 (1.111–1.514)	0.001	1.231 (1.025–1.489)	0.006
TyG	3.179 (2.335–4.364)	<0.001	2.458 (1.737–3.502)	<0.001

Model 1: Unadjusted. Model 2: Adjusted for age, BMI, smoking, hypertension, diabetes, HbA1c, LDL-C, HDL-C, creatinine, leukocytes, uric acid, CRP, and fibrinogen.

SIRI, systemic inflammation response index; TyG, triglyceride-glucose index; OR, odds ratio; CI, confidence interval.

We divided participants into four groups based on the median values of SIRI (0.961) and TyG (8.620) to assess their association with severe stenosis (Table 3). The groups were: low SIRI + low TyG, low SIRI + high TyG, high SIRI + low TyG, and high SIRI + high TyG, with the low SIRI + low TyG group as the reference. The results showed that in Model 1, the combination of high SIRI + high TyG had the strongest association with severe stenosis, with an OR of 7.002 (95% CI: 4.971–9.955, *P* < 0.001). In Model 2, after adjusting for confounders, the OR for high SIRI + high TyG was 4.912 (95% CI: 3.185–7.649, *P* < 0.001). Additionally, we conducted an interaction analysis between SIRI and TyG, which showed that their multiplicative interaction did not yield significant results. Subsequently, we performed an additive interaction analysis (Table 4), and the results were as follows: RERI = 1.947 (95% CI: 0.208–4.103), API = 0.396 (95% CI: 0.042–0.616), and S = 1.991 (95% CI: 1.067–4.430). These results indicate that the additive interaction effect between SIRI and TyG is significantly positively correlated with the occurrence of severe stenosis, and the interaction effect enhances the combined influence of both factors.

**Table 3:** OR (95% CI) of different SIRI and TyG levels for severe stenosis.

Variable	Model 1		Model 2	
	OR (95% CI)	*P* value	OR (95% CI)	*P* value
Severe stenosis				
Low SIRI + Low TyG	ref		ref	
Low SIRI + High TyG	1.830 (1.258–2.665)	0.002	1.474 (0.965–2.252)	0.072
High SIRI + Low TyG	2.843 (1.962–4.140)	<0.001	2.487 (1.601–3.895)	<0.001
High SIRI + High TyG	7.002 (4.971–9.955)	<0.001	4.912 (3.185–7.649)	<0.001

Model 1: Unadjusted. Model 2: Adjusted for age, BMI, smoking, hypertension, diabetes, HbA1c, LDL-C, HDL-C, creatinine, leukocytes, uric acid, CRP, and fibrinogen.

SIRI, systemic inflammation response index; TyG, triglyceride-glucose index; OR, odds ratio; CI, confidence interval.

**Table 4:** Additive interaction of SIRI and TyG for severe stenosis.

Measure	Estimate	95% CI
RERI	1.947	0.208–4.103
API (%)	0.396	0.042–0.616
S	1.991	1.067–4.430

Adjusted for age, BMI, smoking, hypertension, diabetes, HbA1c, LDL-C, HDL-C, creatinine, leukocytes, uric acid, CRP, and fibrinogen.

RERI, relative excess risk due to interaction; API, attributable proportion due to interaction; S, synergy index.

### Predictive capacity and incremental effect of SIRI and TyG on severe stenosis

**Figure 1:**
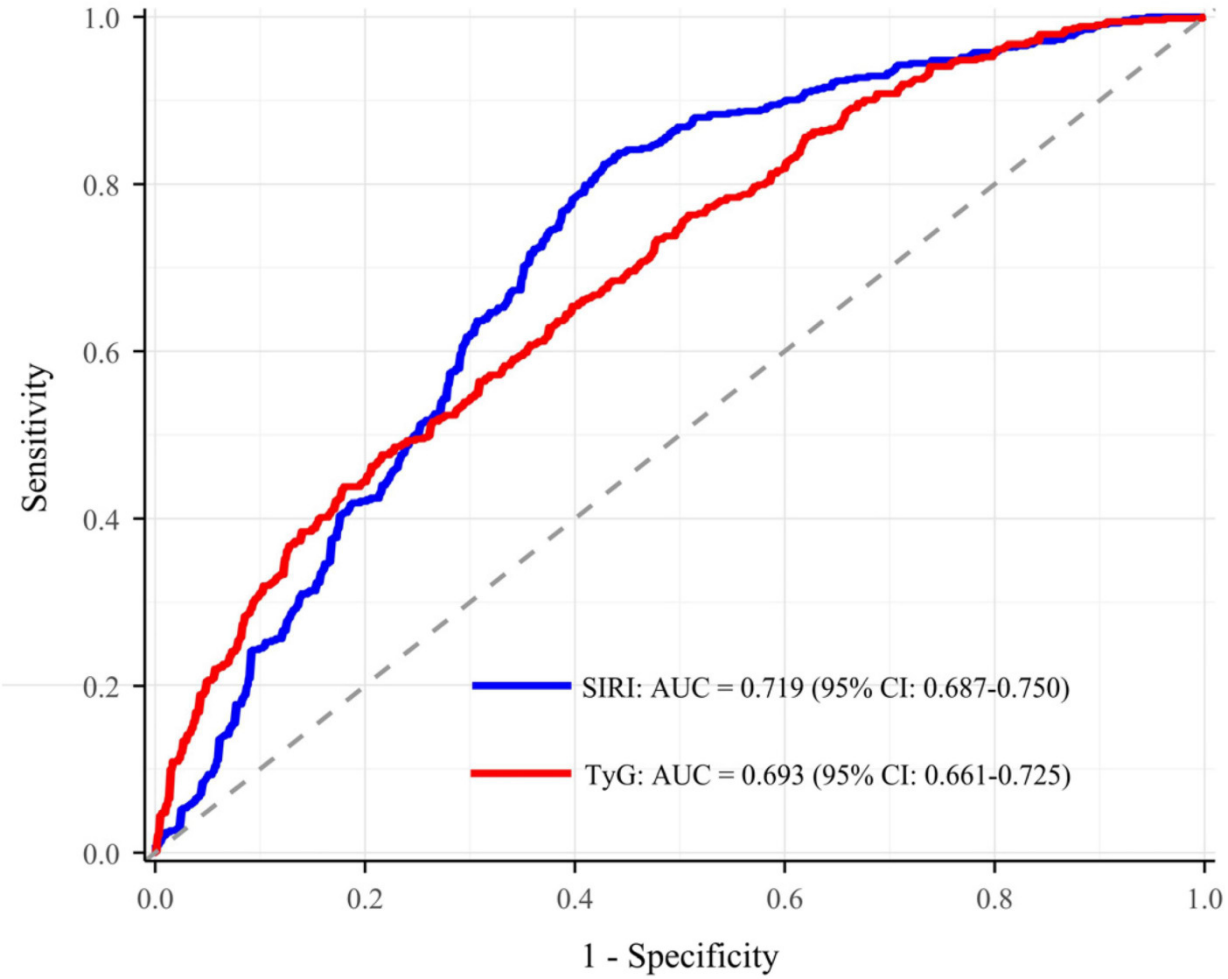
ROC curves for SIRI and TyG. SIRI, systemic inflammation response index; TyG, triglyceride-glucose index; AUC, area under the curve; CI, confidence interval.

**Figure 2:**
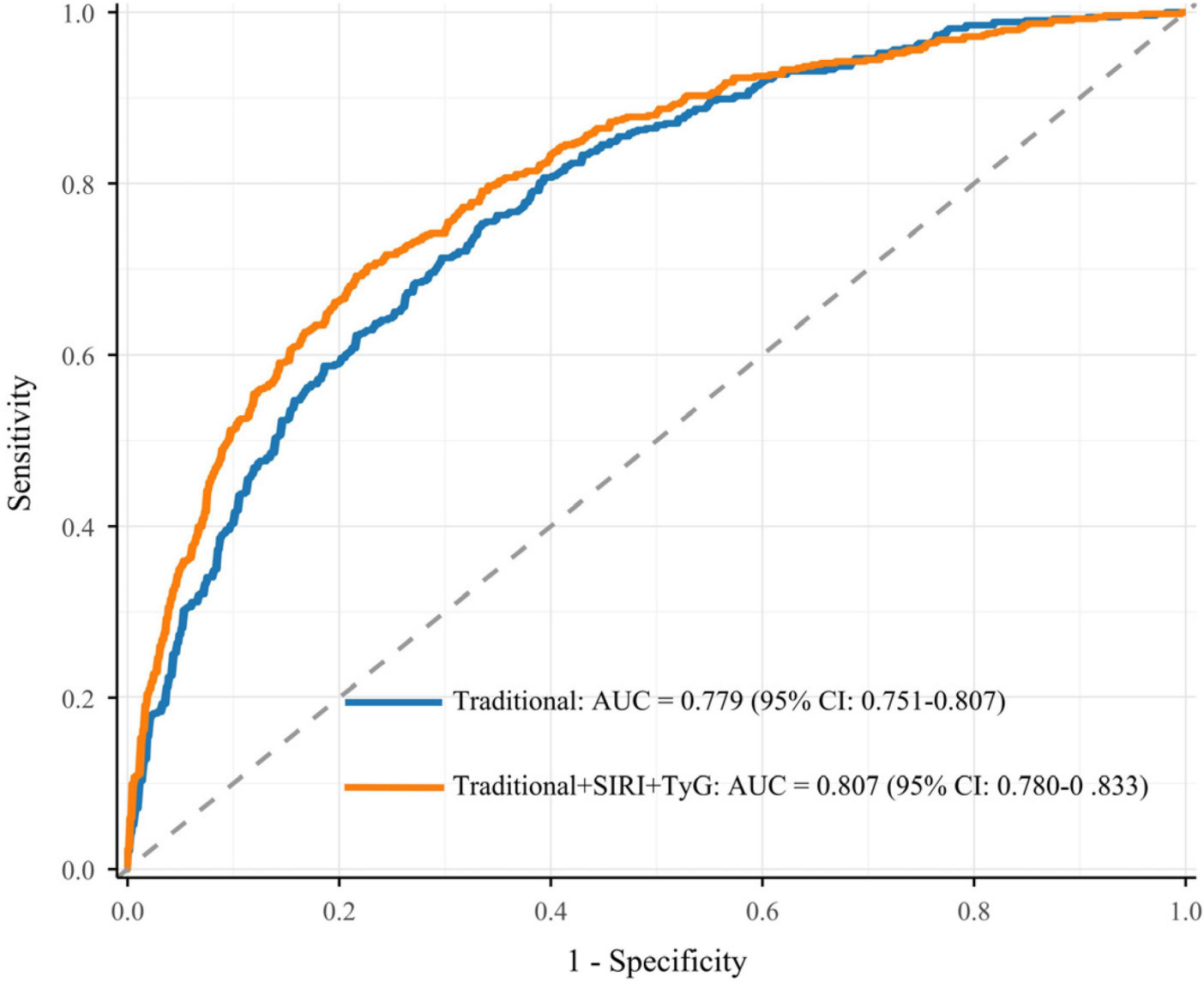
Incremental effect of SIRI and TyG. Traditional risk model included age, BMI, smoking, hypertension, diabetes, HbA1c, LDL-C, HDL-C, creatinine, leukocytes, uric acid, CRP, and fibrinogen. SIRI, systemic inflammation response index; TyG, triglyceride-glucose index; AUC, area under the curve; CI, confidence interval.

In the analysis of severe stenosis risk, we constructed ROC curves and calculated the AUC (Figure 1). The AUC for SIRI was 0.719 (95% CI: 0.687–0.750), and the AUC for TyG was 0.693 (95% CI: 0.661–0.725). Based on the Youden index, the optimal cutoff value for SIRI was 0.433 (sensitivity: 0.824, specificity: 0.573), and for TyG was 0.578 (sensitivity: 0.476, specificity: 0.784) (Supplementary Table S2). Additionally, we assessed the predictive performance of the traditional risk model and the model with the addition of SIRI and TyG. The traditional risk model included age, BMI, smoking, hypertension, diabetes, HbA1c, LDL-C, HDL-C, creatinine, leukocytes, uric acid, CRP, and fibrinogen. The AUC for the traditional risk model was 0.779 (95% CI: 0.751–0.807); when SIRI and TyG were added, the AUC increased to 0.807 (95% CI: 0.780–0.833) (Figure 2). Compared to the traditional risk model, the inclusion of SIRI and TyG significantly improved predictive performance, with a NRI of 0.334 (*P* < 0.001) and an IDI of 0.036 (*P* < 0.001) (Table 5).

**Table 5:** Incremental effect of siri and tyg on predicting severe stenosis.

Measure	Effect Estimate	95% CI	*P* value
NRI	0.334	0.213–0.455	<0.001
IDI	0.036	0.024–0.049	<0.001

NRI, net reclassification improvement; IDI, integrated discrimination improvement; CI, confidence interval.

### Mediation analyses

**Figure 3:**
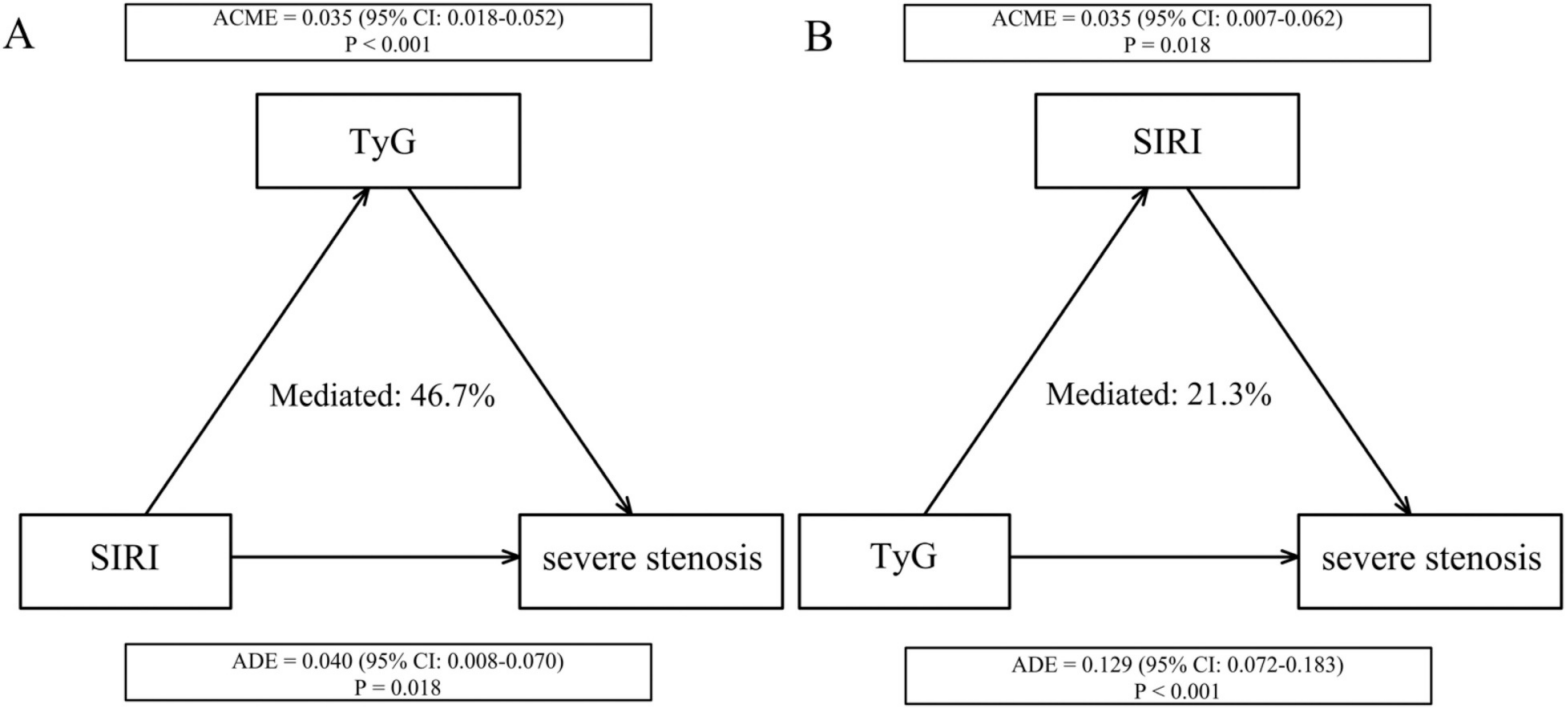
Reciprocal mediation effects of SIRI and TyG on severe stenosis. Adjusted for age, BMI, smoking, hypertension, diabetes, HbA1c, LDL-C, HDL-C, creatinine, leukocytes, uric acid, CRP, and fibrinogen. **(A)** SIRI affects severe stenosis with TyG as a mediator; **(B)** TyG affects severe stenosis with SIRI as a mediator. SIRI, systemic inflammation response index; TyG, triglyceride-glucose index; CI, confidence interval; ACME, average causal mediation effect; ADE, average direct effect.

We evaluated the reciprocal indirect statistical associations of SIRI and TyG in their relationship with severe stenosis (Figure 3). In the first pathway (SIRI→TyG→stenosis), the indirect effect of SIRI on severe stenosis through TyG was significant (ACME: 0.035, 95% CI: 0.018–0.052, *P* < 0.001), accounting for 46.7% of the total effect. The direct effect of SIRI on severe stenosis remained significant (ADE: 0.040, 95% CI: 0.008–0.070, *P* = 0.018). In the reverse pathway (TyG→SIRI→stenosis), the indirect effect of TyG through SIRI was also significant (ACME: 0.035, 95% CI: 0.007–0.062, *P* = 0.018), accounting for 21.3% of the total effect, with a significant direct effect (ADE: 0.129, 95% CI: 0.072–0.183, *P* < 0.001). These findings indicate that SIRI and TyG exhibit significant reciprocal indirect statistical associations in their relationship with severe stenosis.

### Sensitivity analysis using Gensini tertiles

To assess whether the associations of SIRI and TyG with coronary stenosis severity were robust to the median-based dichotomization, we performed a sensitivity analysis in which patients were classified into Gensini score tertiles (Tertile 1: <26, Tertile 2: 26–37, Tertile 3: ≥38). Ordinal logistic regression with both SIRI and TyG entered simultaneously, adjusting for the same covariates as in Model 2, showed that both SIRI (OR: 1.371, 95% CI: 1.181–1.591, *P* < 0.001) and TyG (OR: 1.491, 95% CI: 1.181–1.883, *P* < 0.001) remained independently and significantly associated with higher Gensini tertiles (Supplementary Table S3). These findings are fully consistent with the primary analysis and confirm the robustness of the observed associations across an alternative, more granular classification of stenosis severity.

## Discussion

This study evaluated the significance of SIRI and TyG in coronary artery stenosis, with the following key findings: (1) After adjusting for potential confounders, both higher SIRI and TyG were independently associated with severe stenosis, and an additive interaction between the two was observed; (2) Incorporating SIRI and TyG into the traditional risk model significantly enhanced the predictive value for severe stenosis; (3) TyG mediated 46.7% of the relationship between SIRI and severe stenosis, while SIRI mediated 21.3% of the relationship between TyG and severe stenosis.

Inflammation is a key driver in the formation and progression of atherosclerosis (26). White blood cells, as immune cells, play a crucial role in inflammatory diseases. Recently, the SIRI, which integrates neutrophil, monocyte, and lymphocyte counts, has gained significant attention as an inflammation marker. Compared to traditional two-parameter markers, SIRI provides a more comprehensive assessment of inflammation and immune function, contributing to a deeper understanding of the immune-inflammatory environment. Previous studies have shown that SIRI can serve as a potential biomarker for predicting positive fractional flow reserve, an important method for assessing coronary artery stenosis hemodynamics (27). Sun et al. found that in patients with acute myocardial infarction, elevated SIRI is an independent predictor of the severity of coronary artery disease (28). He et al. demonstrated a significant positive correlation between SIRI and the Gensini score for coronary artery stenosis, and after adjusting for traditional cardiovascular risk factors, the risk of severe coronary artery disease was significantly higher in individuals with elevated SIRI, suggesting an independent role for the inflammatory state in the progression of atherosclerotic lesions (29). The mechanism by which SIRI influences coronary artery stenosis can be understood in the context of immune cell involvement in the pathological process of atherosclerosis. Neutrophils and monocytes are the primary inflammatory effector cells in plaque formation, involved in key processes such as the release of inflammatory mediators, endothelial cell damage, and immune cell infiltration (30). Neutrophils are active in chronic inflammatory environments, and the chemokines and cytokines they release can promote the migration of monocytes to the lesion site and activate local immune responses. Neutrophils also exacerbate endothelial damage through the generation of reactive oxygen species and proteases, further promoting plaque progression and instability (13, 31). These inflammatory processes are critical in worsening vascular narrowing. Upon entering the vascular wall, monocytes differentiate into macrophages, further promoting the inflammatory cascade and accelerating plaque formation (32, 33). Conversely, lymphocytes play a regulatory role in maintaining immune homeostasis, and their reduction is often associated with an imbalance in the inflammatory response and enhanced pro-inflammatory responses, which may accelerate atherosclerosis by promoting inflammatory cell infiltration and inhibiting anti-inflammatory reactions (34, 35). In this study, after adjusting for risk factors, logistic regression analysis revealed a significant correlation between SIRI and severe coronary artery stenosis (OR: 1.231, 95% CI: 1.025–1.489, *P* = 0.006). This association suggests that SIRI holds potential for predicting the degree of coronary artery stenosis, aiding in the early diagnosis and timely intervention of CAD. From an overall inflammatory burden perspective, the association between high SIRI and coronary artery stenosis may be mediated by the release of inflammatory cytokines by neutrophils and monocytes, leading to lymphocyte apoptosis.

IR is one of the major risk factors for cardiovascular disease, acting in concert with other risk factors such as hyperglycemia, dyslipidemia, and hypertension to accelerate the onset and progression of atherosclerosis. Studies have shown that IR plays a significant role through endothelial dysfunction. In the IR state, endothelial cells exhibit reduced responsiveness to insulin, leading to impaired vasodilation, increased oxidative stress, and promoting the formation of vascular stenosis (36, 37). Furthermore, IR enhances local chronic inflammation by activating pro-inflammatory cells and releasing inflammatory cytokines, which are key components in the formation and development of atherosclerotic plaques (38). This study also explored the potential mediating role of IR in the relationship between high SIRI and coronary artery stenosis. A distinctive feature of this study is the demonstration of significant reciprocal indirect statistical associations between SIRI and TyG in relation to coronary stenosis severity. TyG accounted for 46.7% of the SIRI-stenosis association, while SIRI accounted for 21.3% of the TyG-stenosis association. The larger proportion observed for the SIRI stenosis pathway is biologically plausible. Pro-inflammatory cytokines such as TNF-α and IL-6 can directly impair insulin signaling via serine phosphorylation of insulin receptor substrate-1, promote adipose tissue lipolysis, and reduce endothelial nitric oxide bioavailability, all contributing to systemic IR. Through these mechanisms, inflammation may amplify metabolic disturbance, which in turn accelerates plaque progression. Conversely, IR can sustain a pro-inflammatory state via hyperglycemia-induced oxidative stress, advanced glycation end-products, and NF-*κ*B activation, though the magnitude of this feedback may be smaller than the primary inflammatory drive in elderly patients with established coronary atherosclerosis. IR is commonly accompanied by dyslipidemia, characterized by elevated triglycerides and high blood glucose levels. These changes lead to lipid metabolism disorders and promote the accumulation of oxidized low-density lipoprotein (oxLDL). The deposition of oxLDL not only enhances endothelial cell adhesion and chemotaxis of immune cells but also activates monocytes and macrophages, promoting their infiltration into the arterial intima, thereby exacerbating the formation and progression of atherosclerotic plaques (39). Moreover, IR also affects vascular smooth muscle cell function, prompting phenotypic transformation and migration, which increases plaque instability and arterial narrowing (40). Based on these mechanisms, the TyG, as a convenient marker for IR, has been shown to be closely associated with the severity of coronary artery stenosis. The TyG not only reflects metabolic abnormalities but also explains the role of IR in coronary artery disease through its relationship with inflammation, vascular function, and lipid deposition. In the study by Thai et al., a TyG greater than 10 was significantly associated with coronary artery stenosis over 70%, with a sensitivity of 57% and specificity of 75% (41). In adults without coronary artery calcification (CAC) at baseline, the TyG has been identified as an independent predictor of CAC progression (42). Research on non-ST elevation acute coronary syndrome patients indicates that the TyG is independently associated with both the number of coronary stenoses and the SYNTAX score (43). Previous meta-analyses have shown that individuals with a higher TyG have a higher prevalence and severity of coronary artery disease (44).

## Limitations

This study has several limitations. First, as a retrospective cross-sectional study, it cannot fully elucidate the causal relationship between SIRI, TyG, and the severity of coronary artery stenosis. Future prospective cohort studies are needed to confirm our findings and provide a clearer understanding of their causal relationship. Second, this study specifically enrolled patients aged 60 years and above. This age restriction was adopted for two primary reasons. First, the prevalence and severity of coronary atherosclerosis increase substantially after the sixth decade of life, making this age group a clinically relevant, high-burden population for investigating novel risk stratification markers. Second, both systemic inflammation and IR are significantly influenced by age-related physiological changes, including immunosenescence and alterations in metabolic regulation. By restricting the age range, we aimed to minimize the confounding effect of age on the associations between SIRI, TyG, and coronary stenosis severity. Nevertheless, we acknowledge that this restriction inevitably limits the applicability of our findings to younger CAD patients, in whom the roles of SIRI and TyG in coronary stenosis severity may differ due to variations in baseline inflammatory status, insulin sensitivity, and cardiovascular risk factor profiles. Furthermore, age-related changes in immune function and metabolic regulation may modulate the interactions between inflammation and IR in ways that are not generalizable across all age groups. Future multicenter prospective studies with broader age ranges are warranted to validate our findings and explore potential age-specific thresholds for SIRI and TyG in predicting coronary stenosis severity. Caution should be exercised when applying these results to other populations. Third, all participants were recruited from a single tertiary hospital in Cangzhou, Hebei Province, China. The demographic characteristics, lifestyle factors, cardiovascular risk factor profiles, and genetic backgrounds of this population may differ from those of populations in other geographic regions, both within China and internationally. Additionally, clinical practices, including thresholds for coronary angiography and revascularization strategies, may vary across institutions, potentially influencing the observed associations. This single-center design, combined with the age restriction, limits the external validity and generalizability of our findings. Fourth, although several confounding factors were adjusted for in this study, unmeasured residual confounders may still exist. To minimize the impact of lipid-lowering medications, patients with current or recent (within 3 months prior to admission) use of lipid-lowering medications (including statins, ezetimibe, PCSK9 inhibitors, or fibrates) were excluded based on our exclusion criteria. Medication history was obtained through review of electronic medical records and patient self-report at the time of admission. However, this approach may be subject to recall bias and incomplete documentation, potentially leading to misclassification of medication exposure. In addition, due to the inherent limitations of a retrospective study, information on other medications, such as antidiabetic drugs and antiplatelet therapy, was not comprehensively collected. These medications may influence inflammation, IR, and cardiovascular outcomes, thereby representing a potential source of residual confounding that could not be addressed in this study. Furthermore, although patients with recent use of lipid-lowering medications were excluded, detailed lifetime medication history was not systematically captured. Residual influences of prior lipid-lowering therapy on baseline lipid profiles and inflammatory markers cannot be completely ruled out. Furthermore, the exclusion of patients with recent lipid-lowering medication use may have resulted in a selected population with less advanced atherosclerosis or less aggressive lipid management, potentially limiting the generalizability of our findings to patients actively receiving lipid-lowering therapy in routine clinical practice. Fifth, the mediation analysis in this study was performed on cross-sectional data, with SIRI, TyG, and the Gensini score all assessed at a single time point. Mediation analysis conventionally requires a clear temporal sequence(exposure → mediator → outcome) to support causal inference. In the absence of longitudinal data, the temporal relationships among SIRI, TyG, and coronary stenosis severity cannot be established. Therefore, the results of the mediation analysis should be interpreted as statistical associations rather than evidence of biological mediation. The proportions reported reflect the partitioning of statistical effects and do not imply confirmed causal pathways. Future prospective studies with repeated measurements over time are needed to validate the potential mediating roles of these indices in the progression of coronary atherosclerosis. Sixth, the TyG index was used as a surrogate marker for insulin resistance because fasting insulin levels were not available in this retrospective dataset. Consequently, the Homeostasis Model Assessment of Insulin Resistance (HOMA-IR) could not be calculated for comparison. Although the TyG index has been validated as a reliable alternative to HOMA-IR, the absence of a direct insulin resistance measure may have introduced measurement error or attenuated the observed associations. Future studies incorporating fasting insulin measurements are warranted to validate these findings. Additionally, the Gensini score was used to assess the severity of coronary artery stenosis in this study. While it primarily reflects plaque burden, it does not account for factors such as coronary artery bifurcation, calcification, and deformation, which may limit the comprehensive evaluation of coronary artery stenosis severity. Finally, the median Gensini score (30) was used as the cutoff to define severe stenosis in this study, a threshold that lacks clinical validation. Given that different studies have adopted varying Gensini score cutoffs, the median-based definition may limit the comparability of our findings with those of other studies and reduce its direct applicability in clinical practice. Future multicenter prospective studies involving diverse geographic populations and broader age ranges are warranted to validate our findings and establish more generalizable conclusions.

## Conclusion

This study found that SIRI and TyG are important indicators for predicting the risk of coronary artery stenosis. The combined use of both indicators demonstrates strong predictive power, and they interact with each other and exhibit significant reciprocal indirect statistical associations.

## Data Availability

The original contributions presented in the study are included in the article/Supplementary Material, further inquiries can be directed to the corresponding author.
