# Supplementary material for: Association of systemic inflammation response index and triglyceride-glucose index with the severity of coronary artery stenosis in elderly patients: a retrospective cross-sectional study
Source: Front Cardiovasc Med. 2026 May 18;13:1809166. doi: 10.3389/fcvm.2026.1809166 (PMC13222835; doi:10.3389/fcvm.2026.1809166)
Supplement: Supplementary file 1 [file Table1.docx]

**Supplementary Table S1. Spearman correlation coefficients between SIRI, TyG, and continuous Gensini score**

| Variable | ρ | 95%CI | P value |
| --- | --- | --- | --- |
| SIRI | 0.435 | (0.384-0.483) | <0.001 |
| TyG | 0.347 | (0.292-0.400) | <0.001 |

Abbreviations: SIRI: systemic inflammation response index, TyG: triglyceride-glucose index, CI: confidence interval.
