# Supplementary material for: Association of systemic inflammation response index and triglyceride-glucose index with the severity of coronary artery stenosis in elderly patients: a retrospective cross-sectional study
Source: Front Cardiovasc Med. 2026 May 18;13:1809166. doi: 10.3389/fcvm.2026.1809166 (PMC13222835; doi:10.3389/fcvm.2026.1809166)
Supplement: Supplementary file 2 [file Table2.docx]

**Supplementary Table S2. Optimal cutoff values of SIRI and TyG for predicting severe stenosis based on the Youden index**

| Index | AUC | 95% CI | Cutoff | Sensitivity | Specificity | Youden Index |
| --- | --- | --- | --- | --- | --- | --- |
| SIRI | 0.719 | 0.687-0.750 | 0.433 | 0.824 | 0.573 | 1.397 |
| TyG | 0.693 | 0.661-0.725 | 0.578 | 0.476 | 0.784 | 1.260 |

Abbreviations: SIRI: systemic inflammation response index, TyG: triglyceride-glucose index, AUC: area under the curve, CI: confidence interval.
