# Supplementary material for: Association of systemic inflammation response index and triglyceride-glucose index with the severity of coronary artery stenosis in elderly patients: a retrospective cross-sectional study
Source: Front Cardiovasc Med. 2026 May 18;13:1809166. doi: 10.3389/fcvm.2026.1809166 (PMC13222835; doi:10.3389/fcvm.2026.1809166)
Supplement: Supplementary file 3 [file Table3.docx]

**Supplementary Table S3. Sensitivity analysis: ordinal logistic regression of SIRI and TyG with Gensini score tertiles.**

| Variable | OR | 95% CI | P value |
| --- | --- | --- | --- |
| SIRI | 1.371 | 1.181-1.591 | <0.001 |
| TyG | 1.491 | 1.181-1.883 | <0.001 |

Adjusted for age, BMI, smoking, hypertension, diabetes, HbA1c, LDL-C, HDL-C, creatinine, leukocytes, uric acid, CRP, and fibrinogen. Gensini tertiles: Tertile 1 (<26), Tertile 2 (26-37), Tertile 3 (≥38).

Abbreviations: SIRI: systemic inflammation response index, TyG: triglyceride-glucose index, OR: odds ratio, CI: confidence interval.
